# Testing the taxonomy of Dmanisi hominin fossils through dental crown area

**DOI:** 10.1371/journal.pone.0336484

**Published:** 2025-12-03

**Authors:** Victor Nery, Walter Neves, Leticia Valota, Mark Hubbe

**Affiliations:** 1 Research and Dissemination Center in Human Evolution, Institute of Advanced Studies, University of São Paulo, São Paulo, São Paulo, Brazil; 2 Department of Anthropology, The Ohio State University, Columbus, Ohio, United States of America; University of the Witwatersrand, SOUTH AFRICA

## Abstract

The Dmanisi paleoanthropological assemblage from Georgia is among the most debated collections of hominin fossils due to its early age and extreme morphological diversity relative to other *Homo* assemblages. This variability has been interpreted as a result of sexual dimorphism in the *Homo erectus* clade, in which Dmanisi hominins were traditionally classified. However, this hypothesis has been challenged by the proposal that the Dmanisi fossils represent more than one *Homo* species. Taxonomic assessments of the Pleistocene Georgian hominins have focused primarily on craniometric analyses, with fewer studies addressing dental morphology through metric approaches. Considering the value of dental crown area in reconstructing evolutionary relationships, a comparative sample of fossil hominins, consisting of 51 maxillary and 71 mandibular specimens (583 teeth in total), was analyzed using Linear Discriminant Analysis (LDA) to evaluate the diversity in the Dmanisi fossil assemblage. Morphological affinities were examined visually through the first two discriminant functions, and taxonomic relationships were tested via classification analyses based on posterior probabilities. The analyses show a strong association of the D4500-D2600 specimen with australopiths, and of the D2282-D211 and D2700-D2735 specimens with *Homo* species. The sexual dimorphism hypothesis was tested by comparing the ratios of mandibular postcanine dentition of Dmanisi specimens with male and female gorillas and chimpanzees, which suggests that dental crown area of the Pleistocene Georgian hominins could be the product of sexual dimorphism only if they came from species with similar levels of dimorphism than these great apes. We conclude that differences in crown dimensions support the hypothesis of two distinct taxa coexistent at the Dmanisi site, previously proposed to be *Homo georgicus* and *Homo caucasi*. This proposal has important implications for the dispersal of *Homo* out of Africa at the beginning of Pleistocene.

## Introduction

The five hominin fossils recovered from the paleoanthropological site of Dmanisi, located in the Republic of Georgia (Caucasus), have been the subject of intense debate since their first discovery in the 1990s, particularly concerning their taxonomy. Dated to around 1.8 million years ago (Ma), the fossils represent four adults (D2280, D4500-D2600, D3444-D3900, D2282-D211) and one subadult (D2700-D2735) [1–3]. The first specimen excavated at the site was the D211 mandible in 1991, which was later associated with the skull designated as D2282 [4]. The second specimen, the D2280 skull, was excavated in 1999, and it has no associated mandible to date [5]. In 2000, the D2600 mandible was discovered, and in 2005 it was associated with the D4500 skull [6,7]. The D2700 skull was found in 2001, which was later associated with the D2735 mandible [6]. The D3444 skull, found in 2002, represents an almost completely edentulous specimen, and was associated with mandible D3900 in 2003 [8–10].

The taxonomy of the Dmanisi fossil assemblage was primarily analyzed based on cranial morphological affinities [5–7,11]. Compared to well-known hominin species, the five fossils were placed within the *Homo* *erectus* clade, but showed extreme anatomical variability, which was initially interpreted as evidence of high sexual dimorphism in the species [5,8,12–14]. In contrast to the craniometric analysis, there are few Dmanisi metric-focused analyses based on dental morphology [9], which, although recognizing similarities between Dmanisi specimens and australopiths and *Homo habilis*, maintained the species classification of the fossils as *Homo erectus* [9].

The taxonomy of the Dmanisi specimens was challenged by several studies arguing that sexual dimorphism alone could not account for their extreme anatomical variability [11,15–19]. Based on cranial morphological affinities, a large comparative dataset (121 Plio-Pleistocene hominins, represented by 23 linear craniometric dimensions) was used by the authors to propose that the Dmanisi hypodigm comprises two distinct species [11,16]: *Homo georgicus* and *Homo caucasi*. The D4500-D2600 specimen was assigned to *Homo georgicus*, given their closer affinity with australopiths than with the genus *Homo*. In contrast, the D2282-D211 and D2700-D2735 specimens were classified as *Homo caucasi*, due to their similarity with early *Homo*. The possible chronological coexistence of different hominin species at the Dmanisi site has important implications for discussions about early *Homo* dispersion out of Africa, and as such this has been a topic of great interest [11,15,16].

Given the suggestion based on cranial morphology that more than one species is represented in the Dmanisi fossil assemblage [11,15–19], here we tested whether a morphometric analysis of crown area yields a similar result. Leveraging the phylogenetically informative signals retained in posterior dental crown area [20,21], Linear Discriminant Analysis (LDA) was applied to estimate interspecific relationships and to assess the Dmanisi hominin fossil taxonomy based on maxillary and mandibular posterior dentitions.

Morphological traits have been widely used to support the phylogenetic relationships of hominins. We follow a long-standing tradition in paleoanthropology of using dental area as an indicator of evolutionary change [22–28], as well as phylogenetic relationships [29–31]. Geometric morphometrics and molecular data have recently been used to test the goodness-of-fit of hominin specimens to specific phylogenetic hypotheses [32–35], which can incorporate morphological variation in the estimation of species divergence [*e.g.,* 36]. While these methods are able to offer strong model-bound approaches to the estimate of divergence times, crown area is considered a poor source of information for phylogeny, due to the limited number of ratios or qualitatively discrete crown measurements [37–39]. However, teeth dimensions are effective ways to explore taxonomical relationships, since there is a significant portion of the variance in dental dimension that is apportioned to the differences among hominin species, especially when it is analysed within a multivariate framework. As teeth are among the best-preserved skeletal elements in the fossil record and metric analyses are non-destructive, they constitute a powerful source of information about morphological variation and taxonomy [20,21]. The availability of dental remains allows for the creation of large comparative datasets to test taxonomic affinities, as done in this analysis of the Dmanisi specimens.

## Materials and methods

### Dental database

The dental metrics used here are part of a database assembled by the Research and Dissemination Center in Human Evolution of the Institute of Advanced Studies of the University of São Paulo (NPDEH-IEA-USP). The construction of this database was achieved through citation tracking and a systematic review of the literature published in the last three decades, as detailed in a previous publication [40]. The hominin species included in the data span from the Miocene (7 Ma) to the European Upper Paleolithic (30 thousand years ago; ka).

The data reviewed here are based on 22 sources listed in S1 Table, and include mesiodistal (MD) and buccolingual (BL) dimensions of postcanine teeth from the maxilla and mandible of 1,080 specimens (1,572 teeth). These dimensions were taken by different authors using the conventional procedure in biological anthropology of positioning the caliper at the maximum crown length (BL) and width (MD). Although calculating interobserver error is generally recommended in metric-focused dental studies that use published literature like ours [41], this was not possible here due to the amount of sources and the small number of specimens overlapping within these publications. However, it is expected that this error will represent a small portion of the variance, since differences among hominin species are relatively large, especially between genera, and therefore interobserver error should have a small impact on the interpretation of results.

### Classification analyses, dental area calculation, and exclusion criteria

Specimens without a species designation, those preserving only anterior dentition, and those missing more than 50% of the posterior dentition in either the maxilla or the mandible were excluded from the original dental database. From the five Dmanisi hominin fossils included in the data, only the D4500-D2600, D2282-D211, and D2700-D2735 specimens were kept in the analyses. The final dataset of the comparative specimens consists of 241 maxillary teeth from 51 specimens (Table 1) and 342 mandibular teeth from 71 specimens (Table 2). Missing data (5.4% in the maxillae and 3.6% in the mandibles) were estimated using multiple linear regression that considered the existing teeth as predictor variables, following the methodology recently described by us [42].

**Table 1:** Specimen, species, and dental area for the maxillary postcanine dentition.

Specimen	Species	P^3^	P^4^	M^1^	M^2^	M^3^
D4500-D2600	?	118.8	114.4	157.32	204.82	254.28
D2282-D211	?		101.2	162.5	151.2	
D2700-D2735	?		80.5	167.7	164.8	116.6
ARA-VP- 6/1	*Ar. ramidus*	96.25	94.92		166.38	
A.L 191−1	*Au. afarensis*	80.23	79.92	120	152.76	149.34
A.L. 199−1	*Au. afarensis*	84.75	84.36	129.6	162.14	149.34
A.L. 417-1d	*Au. afarensis*	98.28	106.25	160.93	194.04	192.4
A.L. 486−1	*Au. afarensis*	114.66	116.48	184.32	202.64	200.83
A.L. 200-1a	*Au. afarensis*	109.12	102.48	162.26	201.15	215.93
MLD 9	*Au. africanus*	123.3	78	176.9	215.16	192.78
STS 17	*Au. africanus*	117.39	110.04	157.2	200.43	227.2
STS 52a	*Au. africanus*	116.48	139.74	175.26	214.06	187.74
BOU-VP-12/130	*Au. garhi*	182.4	182.4	237.6	254.88	256.88
MH1	*Au. sediba*	100.8	112.53	154.8	176.73	187.53
KNM-ER 1813 A	*H. habilis*	91.53	97.75	156	164.02	147.84
OH 65	*H. habilis*	117.76	117.9	176.8	184.32	154
L894-1	*H. habilis*	111.76	112.24	168.75	164.02	178.35
KNM-ER 1805B	*H. habilis*	98.4	104.4	176.88	172.8	198.56
OH 13	*H. habilis*	95.7	100.05	161.04	176.4	148.03
OH 16	*H. habilis*	113.74	118.58		211.9	
KNM-WT 17400	*P. boisei*	163.08	186	210.6	245.49	230.04
KNM-CH 1	*P. boisei*	141.78	183.28	211.58	268.32	290.5
OH 5	*P. boisei*	185.3	212.4	269.04	361.2	335.98
SK 13	*P. robustus*	130.68	158.55	205.62	249.15	257.25
SK 83	*P. robustus*	118.15	188.71	196.68	221	278.08
DNH 7	*P. robustus*		131.56	169.2	169.36	175.45
TM 1517	*P. robustus*	142.14	163.71	200.02	220.57	233.28
ZHK XIII	*H. erectus*	102.08	73.92	126.48	131.58	117.37
SIN O1	*H. erectus*	92.8	81.03	131.44	142.04	122.5
SIN L2	*H. erectus*	77.7	82.88	125.46	130.56	125.84
Sangiran 17	*H. erectus*	66.93	79.18	152.22	143.36	125.96
Sangiran 4	*H. erectus*	107.52	103.32	156.94	209.44	143.52
PITH B/S1B	*H. erectus*	105.4	104.55	167.28	206.72	151.2
SIN H3	*H. erectus*	103.7	77	126	119	111.36
KNM-WT 15000	*H. erectus*	95.45	94.3	130.98	155.61	
Rabat	*H. erectus*	102	88	144	149.5	
Petralona 1	*H. heidelbergensis*	109.22	96.52	176.4	160.46	143.38
LES1	*H. naledi*	87.2	91.53	127.33	154.88	155.04
SAC 2	*H. neanderthalensis*	74.16	73.14	134.47	116.16	106.4
Sima 5	*H. neanderthalensis*			104.64	86.52	101.52
Tabun c1	*H. neanderthalensis*	73.26	64.68	124.26	121.9	87.87
Cesaire	*H. neanderthalensis*	72.96	69.12	118.72	116.16	114
SHAN 1	*H. neanderthalensis*	61.1	59.52	114.66	105.8	102.35
SPY 2	*H. neanderthalensis*	64.48	65.8	114.13	110.2	131.44
AMUD	*H. neanderthalensis*	74.74	67.2	119.34	118.32	
SHAN 2	*H. neanderthalensis*	70	72.1	134.07	143.51	119.31
SHAN 6	*H. neanderthalensis*	65.34	79.18	135.3	151.04	130
Mislyia	*H. sapiens*	68.62	61.88	127.92	119.31	105.02
Skhul V	*H. sapiens*	75.44	68.62	132.24	125.43	100.57
QFZEH 9	*H. sapiens*	90.3	72	156.24	140.42	132.16
LB1	*H. floresiensis*	61.6	63.19	103.5	100.44	

**Table 2:** Specimen, species, and dental area for the mandibular postcanine dentition.

Specimen	Species	P_3_	P_4_	M_1_	M_2_	M_3_
D4500-D2600	?	120			175.5	154.28
D2282-D211	?	89.2	77.6	163.7	142.7	115.5
D2700-D2735	?	98	71.4	148.2	141.24	
KNM-KP 29286	*Au. anamensis*	122.76	113.49	147.6	200.02	184.32
KNM-KP 47953	*Au. anamensis*	120.32	120.32	165.6	165.6	253.68
A.L. 400-1a	*Au. afarensis*	109.76	109.76	166.32	219	117.6
A.L. 288-1i	*Au. afarensis*	84	86.1	136.4	161.04	173.24
A.L. 333W-32, 60	*Au. afarensis*	113.4	121.6	174.24	211.7	203.06
A.L. 417-1a	*Au. afarensis*	96.12	96.32	147.56	172.92	204.82
A.L. 266−1	*Au. afarensis*	100.58	103.79	151.13	182	216.46
LH 4	*Au. afarensis*	106	114.4	163.8	201.28	231.46
STS 52a	*Au. africanus*	105.3	114.66	167.7	192.96	173.99
STS 52b	*Au. africanus*	105.91	118.58	182.16	205.2	175.36
MLD 18	*Au. africanus*	118	106.2	165.06	204.33	204.48
MLD 40	*Au. africanus*	102.12	109.61	162.44	215.73	210
L75 - 14	*Au. africanus*	137.76	144.78	219.96	255.64	212.91
STS 7	*Au. africanus*	102	119.7	152.4	230.68	236.16
STS 36	*Au. africanus*	126.35	119.7	172.28	250.12	280.36
MH2	*Au. sediba*	74.52	85.36	148.03	177.12	185
MH1	*Au. sediba*			150.65	191.4	202.64
OH 13	*H. habilis*	75.6	88.2	150.8	170.4	182.04
OH 16	*H. habilis*	117.66	111.1	186.88	232.54	227.37
L7 - 125	*P. boisei*	182	221.13	314.16	291.6	269.36
KNM-ER 15930	*P. boisei*		168	186.88	232	273
Peninj 1	*P. boisei*	135.34	219	255.64	288.36	293.02
NATRON	*P. boisei*	124.2	220.4	250.92	281.88	306.44
KNM-ER 3230	*P. boisei*	158.46	239.25	261.8	383.8	338.25
KNM-ER 729	*P. boisei*	156	224	240.25	333	418
DNH 7	*P. robustus*	113.16	129.78	168.84	180.9	206.72
SK 55	*P. robustus*	105.6		197.28	224.51	212.35
SK 23	*P. robustus*	110.4	159.84	224.96	224.96	220.08
TM 1517	*P. robustus*	108.9	144.1	195.36	224.96	234.52
TM 1600	*P. robustus*	128.96	124.26	178.2	226.38	239.89
SK 12	*P. robustus*	140.98	162	193.2	237.8	264.69
SK 34	*P. robustus*	132.08	169.74	207	282.15	289.6
SK 6	*P. robustus*	130	135.3	258.85	289.98	289.85
SK 876	*P. robustus*	117	131.25	196.3	256.7	290.45
DNH 8	*P. robustus*	128.52	153.68	227.65	238.5	309.42
KNM-ER 729A	*P. robustus*	164.56	219	262.4	369	402.8
KNM-WT 15000	*H. erectus*	85.85	85.5	132.98	102.35	
SIN B1	*H. erectus*	80.08	79.2	132.09	142.08	
Rabat	*H. erectus*	90	85.5	143	141.25	137.5
ZKD G1-6	*H. erectus*	97.37	93.5	165	158.75	147.6
SIN D1	*H. erectus*	75.44			146.4	147.62
SIN 16	*H. erectus*	87.4	99.96	180.7	156.25	156.8
KNM-ER 992A	*H. erectus*	99.75	94.6	136.25	167.28	159.72
KNM-ER 992B	*H. erectus*	99.91	95.92	138.43	158.6	164.82
Tigenhif 3	*H. erectus*	82.4	80	153.75	150.06	139.2
Tigenhif 1	*H. erectus*	83.3	80	165	171.6	152.5
Tigenhif 2	*H. erectus*	93.5	99	182	189	167.5
LB6	*H. floresiensis*	63.96	51.59	93	93.1	75.65
LB1	*H. floresiensis*	71.4		99.84	101	94.08
TERN 3	*H. heidelbergensis*	82.72	79.68	134.31	128.76	126.44
TERN 1	*H. heidelbergensis*	78.72	72.75	139.7	152.5	136.85
TERN 2	*H. heidelbergensis*	84.66	86.86	156.94	164.4	144.48
Arago 13	*H. heidelbergensis*	106.92	102.12	172.8	202.34	158.51
LES1	*H. naledi*	78.12	74.62	118.72	141.45	155.61
UW 101–1261	*H. naledi*	73.08	78.3	122.04	135.42	161.13
Sima 5	*H. neanderthalensis*	50.4	47.2	88.35	102	98.94
Tabun c1	*H. neanderthalensis*	64.8	49.3	95.79	113.12	99.84
Cesaire	*H. neanderthalensis*	68.73	53.46	108.07	106.02	118.45
Tabun c2	*H. neanderthalensis*	70.31	67.34	113.12	113.3	119
SHAN 1	*H. neanderthalensis*	68.25	55.68	112.32	116.63	119.34
SPY 2	*H. neanderthalensis*	58.32	53.72	115.54	125.28	119.9
AMUD	*H. neanderthalensis*	68.08	61.06	115.56	113.22	121.68
Kebara 2	*H. neanderthalensis*	68.25	70.56	117.72	118.77	126.1
SHAN 2	*H. neanderthalensis*	68.4	60.06	126.44	135.6	131.04
SHAN 6	*H. neanderthalensis*	66.96	66.43		148.68	156.16
UC 101	*H. sapiens*	55.89	54.78	112.35	115.5	109
Skhul V	*H. sapiens*	66.4	60.68	119.78	126.36	118.17
QFZEH 9	*H. sapiens*	75.44	74.88	158.72	145.2	153.12

The MD and BL dimensions of premolars and molars were used to calculate dental areas by multiplying both dimensions, following the procedure in previous studies [24,26]. The mandibular and maxillary data were kept separated in the analyses to maximize the representation of dental area for fossils in which only the maxillae or the mandible were present. Table 3 presents the sample sizes of teeth used in the analyses by species.

**Table 3:** Number of teeth available for each species in the comparative dataset.

Species	P_3_	P_4_	M_1_	M_2_	M_3_	P^3^	P^4^	M^1^	M^2^	M^3^
Dmanisi hominin fossils	3	2	2	3	2	1	3	3	3	2
*Ar. ramidus*						1	1		1	
*Au. anamensis*	2	2	2	2	2					
*Au. afarensis*	6	6	6	6	6	5	5	5	5	5
*Au. africanus*	7	7	7	7	7	3	3	3	3	3
*Au. garhi*						1	1	1	1	1
*Au. sediba*	1	1	2	2	2	1	1	1	1	1
*H. habilis*	2	2	2	2	2	6	6	5	6	5
*P. boisei*	5	6	6	6	6	3	3	3	3	3
*P. robustus*	11	10	11	11	11	3	4	4	4	4
*H. erectus*	11	10	10	11	9	9	9	9	9	7
*H. heidelbergensis*	4	4	4	4	4	1	1	1	1	1
*H. naledi*	2	2	2	2	2	1	1	1	1	1
*H. neanderthalensis*	10	10	9	10	10	8	8	9	9	8
*H. sapiens*	3	3	3	3	3	3	3	3	3	3
*H. floresiensis*	2	1	2	2	2	1	1	1	1	

Multivariate Linear Discriminant Analysis (LDA) was used to explore the morphological affinities of the D4500-D2600, D2282-D211 and D2700-D2735 specimens with 15 well-accepted hominin species (Tables 1–3). Discriminant functions were calculated for each of the datasets without the inclusion of the Dmanisi specimens, and were used to classify them *a posteriori.* Classification of the Dmanisi hominin fossils was based on the posterior probability of belonging to each of the hominin species in the reference data. To visualize the morphological affinities of the Dmanisi specimens, the scores of the first two discriminant functions were used to create a scatterplot illustrating the morphospace occupied by all the individuals in the analyses. All analyses were conducted in R [43]. LDA was implemented with the MASS package [44]. Data visualization was achieved using the ggplot2 and ggrepel packages [45,46].

### Evaluating the sexual dimorphism hypothesis

As morphological differences in Dmanisi hominin fossils were suggested to be the result of sexual dimorphism, we also tested if the ratios between individual dental size of these fossils falls within the range of sexual dimorphism in extant apes. The ratios of the mandibular postcanine dentition of the three Dmanisi specimens were compared with the ratios between 15 male and 14 female gorillas, and 11 male and 11 female chimpanzees. Only mandibular data were considered because the comparative data used is limited to a sample with only mandibular data. However, it is expected that the size ratios of postcanine teeth between sexes of the great apes will not differ significantly between maxillary and mandibular dentition. The ratios of mandibular postcanine dentition of the Dmanisi specimens compared to gorillas and chimpanzees were represented visually using ggplot2 in R [45].

## Results

Table 4 presents the classification results for the three Dmanisi hominin specimens. In both the maxillary and mandibular dentition, the Dmanisi fossil assemblage shows a very distinctive classification when compared with other hominin species. For the maxillary dentition, the D2282-D211 and D2700-D2735 specimens show their strongest posterior probabilities to species of the genus *Homo* (*Homo habilis* – PP = 0.83 and *Homo sapiens* – PP = 0.59, respectively), and the D4500-D2600 specimen shows strongest classification probabilities with *Australopithecus* species (*Australopithecus africanus* – PP = 0.78). For the mandibular dentition, the primary classification of the Dmanisi specimens was with *Homo erectus*, but with relatively low posterior probability values (0.31 for D4500-D2600, 0.52 for D2700-D2735, and 0.58 for D2282-D211). Similar to the analysis of the maxillary dentition, the second and third highest posterior probabilities of the mandible dentition separate the D4500-D2600 specimen, which is associated with *Australopithecus africanus* (*p *= 0.24), from the D2700-D2735 and D2282-D211 specimens, which are classified as *Homo heidelbergensis* (*p *= 0.22 and *p *= 0.26, respectively; Table 4).

**Table 4:** First three classifications and associated posterior probabilities for the Dmanisi specimens based on the maxillary and mandibular dentitions.

Specimen	First Classification		Second Classification		Third Classification	
	Species	Posterior probability	Species	Posterior probability	Species	Posterior probability
**Maxillary** **dentition**						
D4500- D2600	*Au. africanus*	0.786	*Au. afarensis*	0.183	*Au. sediba*	0.027
D2282- D211	*H. habilis*	0.834	*H. heidelbergensis*	0.087	*H. erectus*	0.036
D2700- D2735	*H. sapiens*	0.59	*H. heidelbergensis*	0.180	*H. habilis*	0.098
**Mandibular dentition**						
D4500- D2600	*H. erectus*	0.317	*Au. africanus*	0.243	*Au. afarensis*	0.240
D2282- D211	*H. erectus*	0.588	*H. heidelbergensis*	0.264	*H. neander-* *thalensis*	0.058
D2700- D2735	*H. erectus*	0.524	*H. heidelbergensis*	0.216	*H. neander-* *thalensis*	0.105

Tables 5 and 6 show the correct classification frequencies of the three Dmanisi specimens to their respective species for the maxillary and mandibular dentitions. The primary classification for the maxillary dentition presents a relatively low rate of correct classification (mean = 54.6%; sd = 41.5%). However, when the classification criteria goes beyond the highest posterior probability alone, very few of the specimens have a probability of association to their own species lower than 0.05. For the maxillary dentition, the frequency of individuals that show posterior probability higher than 0.05 to their own species is 97.8% (sd = 5.9%), which demonstrates that the discriminant functions classification does not reject the hypothesis that the specimens could belong to their own species in almost all individuals, despite the low correct classifications based on the largest posterior probabilities. The results for the mandibular dentition are similar to the maxillary one, with the average frequency of correct classification based on the largest posterior probability equal to 45.5% (sd = 40.6%) and frequency of specimens with posterior probability larger than 0.05 to their own species equal to 99.3% (sd = 2.5%).

**Table 5:** Frequency of correct classifications of the specimens in the comparative database, based on areas of the maxillary postcanine dentition.

Species	N	Correct primary classification		Correct classification for posterior probability > 0.05	
		n	frequency	n	frequency
*Ar. ramidus*	1	0	0	1	1
*Au. afarensis*	5	2	0.4	4	0.8
*Au. africanus*	3	2	0.67	3	1
*Au. garhi*	1	1	1	1	1
*Au. sediba*	1	0	0	1	1
*H. erectus*	9	6	0.67	8	0.89
*H. floresiensis*	1	0	0	1	1
*H. habilis*	6	5	0.83	6	1
*H. heidelbergensis*	1	1	1	1	1
*H. naledi*	1	0	0	1	1
*H. neanderthalensis*	9	9	1	9	1
*H. sapiens*	3	1	0.33	3	1
*P. boisei*	3	3	1	3	1
*P. robustus*	4	3	0.75	4	1

**Table 6:** Frequency of correct classifications of the specimens in the comparative database, based on areas of the mandibular postcanine dentition.

Species	N	Correct primary classification		Correct classification for posterior probability > 0.05	
		n	frequency	n	frequency
*Au. afarensis*	6	3	0.5	6	1
*Au. africanus*	7	6	0.86	7	1
*Au. anamensis*	2	1	0.5	2	1
*Au. sediba*	2	1	0.5	2	1
*H. erectus*	11	10	0.91	11	1
*H. floresiensis*	2	0	0	2	1
*H. habilis*	2	0	0	2	1
*H. heidelbergensis*	4	0	0	4	1
*H. naledi*	2	0	0	2	1
*H. neanderthalensis*	10	10	1	10	1
*H. sapiens*	3	0	0	3	1
*P. boisei*	6	5	0.83	6	1
*P. robustus*	11	9	0.82	10	0.91

**Fig 1:**
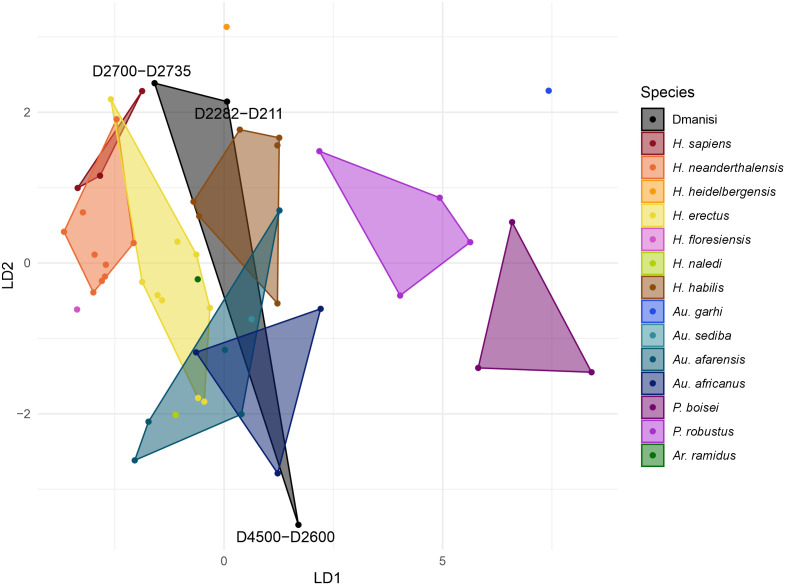
Morphological affinities of Dmanisi compared to other hominin species based on the first two discriminant functions calculated from maxillary dentition areas.

**Fig 2:**
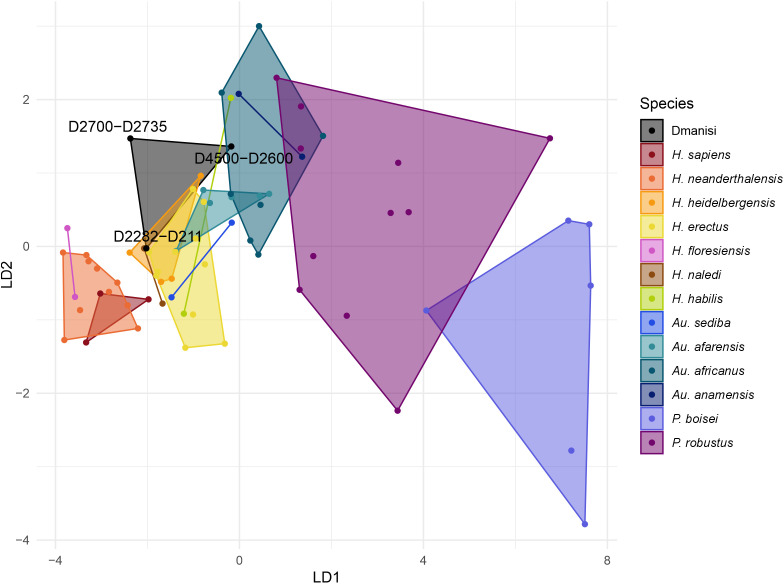
Morphological affinities of Dmanisi compared to other hominin species based on the first two discriminant functions calculated from mandibular dentition areas.

Figs 1 and 2 show the position of the three Dmanisi specimens in the morphospace illustrating their morphological affinities based on the first two discriminant functions calculated from the comparative data. In the analysis of the maxillary dentition (Fig 1), the first discriminant function separates the *Paranthropus* from the *Australopithecus* and *Homo* specimens, and the second discriminant function separates the *Australopithecus* from the *Homo* specimens. The Dmanisi fossils show clearly different morphological affinities to the hominin species in the comparative data. The D4500-D2600 specimen is separated from *Homo* specimens and clearly integrated in the *Australopithecus* morphospace. The D2282-D211 and D2700-D2735 specimens show similar morphological affinities to each other and are well integrated in the morphospace of the genus *Homo*.

In the mandibular dentition analysis (Fig 2), the first discriminant function separates the *Paranthropus* individuals from the other hominins, such as in the maxillary analysis. There is a general chronological association among the *Australopithecus* and *Homo* in this axis as well. The second discriminant function separates *Australopithecus*, with higher values, from *Homo*, with lower values. The three Dmanisi hominin fossils are relatively closer to the early *Homo*, with the D4500-D2600 specimen well integrated in the morphospace of *Australopithecus africanus* and close to *Australopithecus afarensis*. The D2282-D211 specimen is within the range of *Homo ere*ctus and *Homo heidelbergensis*, and the D2700-D2735 specimen is within the early *Homo* range for the first discriminant function, but it is separated from them on the second discriminant function.

**Fig 3:**
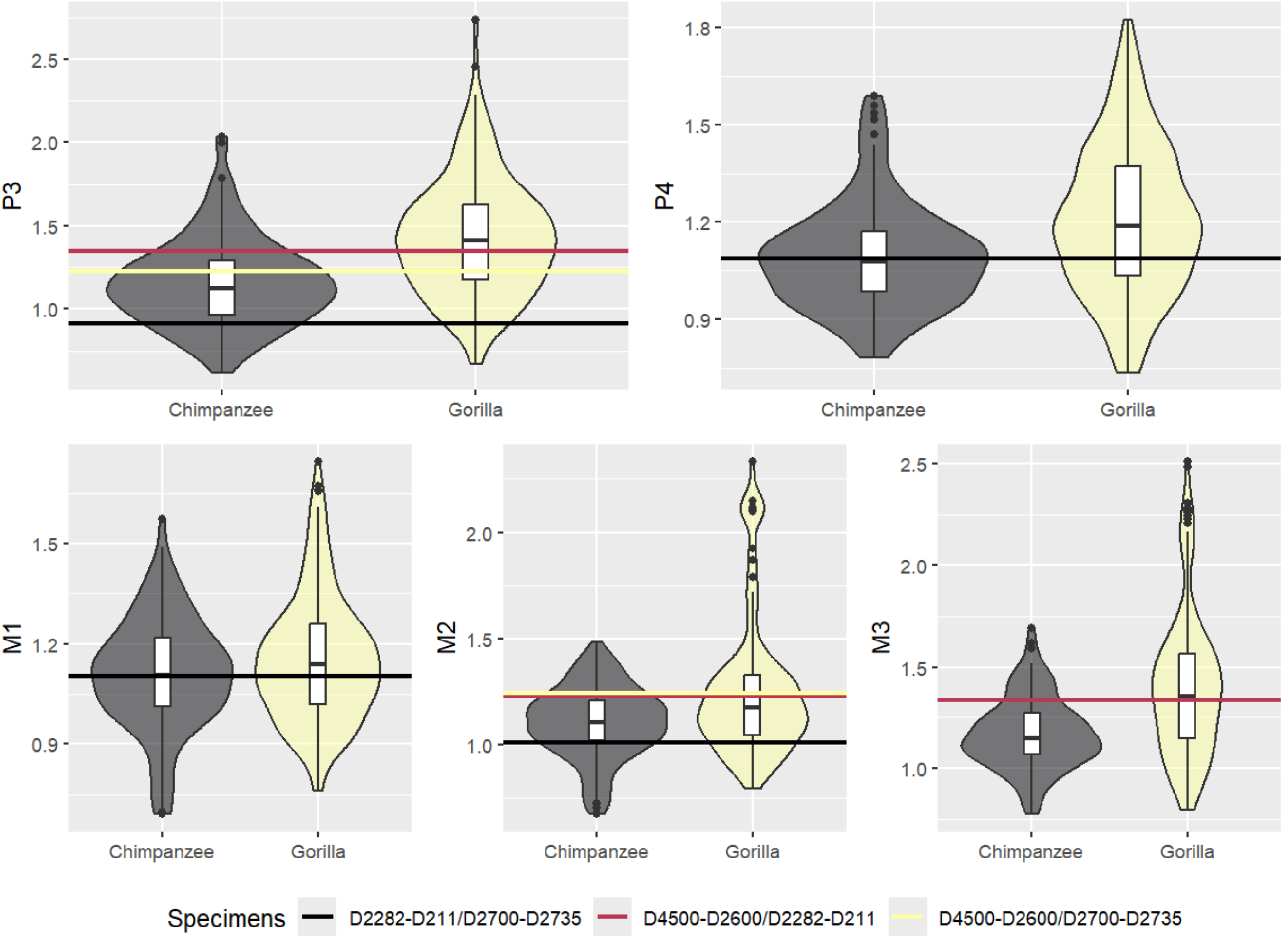
Ratios of mandibular postcanine area of Dmanisi specimens compared to the distribution of male/female size rations in gorillas and chimpanzees.

Fig 3 and Table 7 show the ratios of mandibular postcanine area of the three Dmanini specimens compared to gorillas and chimpanzees, which have greater sexual dimorphism than humans. The Pleistocene Georgian hominins ratios fall within the range of chimpanzees and gorillas for all teeth. For the three teeth available in the larger specimen (D4500-D2600), the area ratio between this specimen and the the two smaller specimens (D2282-D21/ D2700-D2735) is close to the median of gorillas and above the median of chimpanzees. This comparison shows that the size differences between the Dmanisi specimens’ postcanine area falls within the range of these extant apes.

**Table 7:** Mandibular postcanine area for the Dmanisi specimens and comparative summary statistics for gorillas and chimpanzees.

Specimens/ Species	Sex	N	Statistics	P_3_	P_4_	M_1_	M_2_	M_3_
**D4500-D2600**				120			175.5	154.28
**D2282-D211**				89.2	77.6	163.7	142.7	115.5
**D2700-D2735**				98	71.4	148.2	141.24	
**Gorilla**	F	14	Mean	134.56	133.50	178.51	217.51	185.57
			St. dev.	25.46	18.77	22.78	39.42	32.27
			Min	94.08	102.46	135.66	134.4	124.74
			Max	180.48	162.44	225.6	264.48	234.52
**Gorilla**	M	15	Mean	185.91	158.23	202.6	263.09	251.36
			St. dev.	31.31	20.8	20.05	30.45	39.73
			Min	121.26	119.6	171.99	211.2	186.66
			Max	257.4	186.96	236.6	313.24	313.23
**Chimpanzee**	F	11	Mean	79.88	70.48	104.10	110.66	96.80
			St. dev.	12.7	9.05	10.21	8.44	10.86
			Min	54.78	52.5	85.44	97.92	77.43
			Max	103.2	85.44	117.16	125.28	112.36
**Chimpanzee**	M	11	Mean	90.24	75.89	114.50	121.93	112.3
			St. dev.	14.12	5.45	13.70	16.98	11.33
			Min	64.05	66.88	81.0	84.66	87.22
			Max	111.28	83.43	134.4	145.14	131.08

## Discussion

### The taxonomy of Dmanisi specimens

The comparative analysis of the dental crown area of the postcanine teeth from the three Dmanisi specimens included in our analyses support their classification in more than one species, as recently proposed [11,15–19]. The classification results (Tables 4–6 and Figs 1 and 2) demonstrate that the posterior dentition of these fossils is extremely diverse when compared to well-characterized hominin species. The D4500-D2600 specimen showed strong similarity to australopiths, while D2282-D211 and D2700-2735 specimens demonstrated stronger affinity with early *Homo*. This pattern is observed in both dental arcades, but the differentiation is more evident in the maxillary dentition, in which the larger specimen shows a very distinct classification pattern, based on posterior probabilities, and a clearly different position in the morphospace (Fig 1).

The differences among the Dmanisi specimens have been traditionally explained as a product of sexual dimorphism [5,8,9,12–14]. We show that these differences fall within the range of chimpanzees and gorillas, suggesting that they could be the product of sexual dimorphism if the Dmanisi hominin fossils came from a species with a similar level of sexual dimorphism to these great apes. These results indicate that we are unable to reject the hypothesis that Dmanisi specimens represent males and females of a single species based on crown area ratios. However, despite these results, we argue that the differences among the Dmanisi specimens are more parsimoniously explained by the existence of more than one species in the Dmanisi site. The strong association of the D4500-D2600 specimen with *Australopithecus* species is not only a function of the larger size of the teeth, but also that this specimen has a relative large M^3^ (Table 1), which goes in opposition to the trend in later *Homo* of showing smaller third molars [37]. This degree of separation can hardly be explained by sexual dimorphism alone, as it is not just a function of the size of the teeth. Moreover, while the area ratio between the largest Dmanisi specimen and the two smaller ones falls within the range of great apes, it still would represent more morphological diversity than the one observed among other *Homo* species, as well illustrated in Fig 1. This variety contrasts with the *Homo erectus* dental classification proposed initially for the Dmanisi hominin fossils [9], even though a delayed formation in the posterior dentition has recently been shown in D2700-D2735 specimen [47]. Therefore, similar to the results of cranio-morphological classification [11,16], the postcanine dental crown area of the three Dmanini specimens analysed here supports the taxonomic classification of the D4500-D2600 specimen as *Homo georgicus*, and the classification of the D2282-D211 and D2700-D2735 specimens as *Homo caucasi*.

### Phylogenetic history of Dmanisi

Although our analyses did not formally test the phylogenetic history of the Pleistocene Georgian hominins, the proposal of more than one species in the Dmanisi fossil assemblage has implications for the dispersal of the genus *Homo* out of Africa in the beginning of the Pleistocene [15,16,48]. It is traditionally accepted that the *Homo erectus* migration started in Kenya (Turkana) around 1.89 Ma, reached Georgia (Dmanisi) around 1.77 Ma, continued into eastern Eurasia (Yuanmou) around 1.7 Ma, and finally arrived to Indonesia (Sangiran) by ~1.57 Ma [49,50]. However, the speciation events that led to the evolution of more than one species in Dmanisi requires that lineages were separated for long periods after leaving Africa, and were likely also evolving in response to different selective environments.

In recent years, several hypotheses have been proposed to explain the motivations behind the *Homo erectus* dispersal out of Africa [51,52]. Brain expansion has been suggested as the primary driver of *Homo* expansion, as increased cognitive capacity associated with efficient bipedal locomotion would allow *Homo erectus* to expand into new ecological niches. Cultural exclusion, which suggests that the emergence of Acheulean technology may have displaced Oldowan tool-using populations, has also been suggested as a main driver for expansion. Other hypotheses have been ecological in nature. For instance, it has been proposed that shifts in African fauna led associated consumers, including hominins, to move toward Eurasia [51,52].

If the Dmanisi specimens cannot be taxonomically grouped with *Homo erectus* [11,16], it raises the possibility that early *Homo* evolution had multiple episodes of cladogenesis, where some of them may have started in Africa, and others outside Africa. Of particular interest to this discussion is the high similarity between the D4500-D2600 specimen and australopiths, which suggests either a retention of the ancestral dental proportions of australopiths in Dmanisi, or an evolutionary convergence after the initial differentiation of early *Homo.* With the evidence available, it is not possible to properly evaluate if *Homo georgicus* and *Homo caucasi* evolved from *Homo erectus* ancestors, or if they evolved from australopith-like ancestors, but alternative scenarios are worth exploring and considering as new early *Homo* fossils are discovered in Asia.

Recent discoveries have been published and support alternative scenarios of *Homo* migration out of Africa. The new ^26^Al/^10^Be ages from Yuanmou and Sangiran suggest that *Homo erectus* may have reached the farthest regions of Asia as early as 1.8 Ma [53,54], which contradicts the traditional route of dispersal [49,50]. Moreover, regions such as the Middle East and the southern fringes of Eurasia may have been more ecologically and biogeographically integrated with the African landscape than traditionally assumed, potentially creating favorable climatic conditions for the development of new hominin species [55]. Recent discoveries of Oldowan tools and associated cut marks in Jordan and Romania, respectively, predate the arrival of *Homo erectus* to these regions, offering further support for the presence of earlier hominin species in the north of or even outside of Africa [16,56,57]. The diversity of the Dmanisi hominin fossils, and the possibility that they represent more than one species, adds to this discussion demonstrating that a revision of our current models for the expansion of *Homo* out of Africa is required.

## Conclusion

The postcanine dental crown area analysis of the Dmanisi hominin fossils (D4500-D2600, D2282-D211, and D2700-D2735) supports the hypothesis of distinct species coexisting temporally at the site (*Homo caucasi* and *Homo georgicus*). This possibility challenges the prevailing model of *Homo erectus* migration out of Africa by suggesting that the evolution of early *Homo* probably involved multiple cladogenesis events that were likely associated with different expansion processes and responses to diverse selective environments.

## Supporting information

S1 Table Publication sources for NPDEH’s dental database. Adapted from our team of research’s recent publication [40].
(XLSX)

## References

[1] Gabunia L, Vekua A. A Plio-Pleistocene hominid from Dmanisi, East Georgia, Caucasus. Nature. 1995;373(6514):509–12. doi: 10.1038/373509a0 7845461

[2] Berger LR, Tobias PV. A chimpanzee-like tibia from Sterkfontein, South Africa and its implications for the interpretation of bipedalism in Australopithecus africanus. J Hum Evol. 1996;30(4):343–8. doi: 10.1006/jhev.1996.0027

[3] Rosas A, Bermúdez De Castro JM. On the taxonomic affinities of the Dmanisi mandible (Georgia). Am J Phys Anthropol. 1998;107(2):145–62. doi: 10.1002/(sici)1096-8644(199810)107:2<145::aid-ajpa2>3.0.co;2-u 9786330

[4] Gabounia L, de Lumley M-A, Vekua A, Lordkipanidze D, de Lumley H. Découverte d’un nouvel hominidé à Dmanissi (Transcaucasie, Géorgie). Comptes Rendus Palevol. 2002;1(4):243–53. doi: 10.1016/s1631-0683(02)00032-5

[5] Gabunia L, Vekua A, Lordkipanidze D, Swisher CC 3rd, Ferring R, Justus A, et al. Earliest Pleistocene hominid cranial remains from Dmanisi, Republic of Georgia: taxonomy, geological setting, and age. Science. 2000;288(5468):1019–25. doi: 10.1126/science.288.5468.1019 10807567

[6] Vekua A, Lordkipanidze D, Rightmire GP, Agusti J, Ferring R, Maisuradze G, et al. A new skull of early Homo from Dmanisi, Georgia. Science. 2002;297(5578):85–9. doi: 10.1126/science.1072953 12098694

[7] Lordkipanidze D, Ponce de León MS, Margvelashvili A, Rak Y, Rightmire GP, Vekua A, et al. A complete skull from Dmanisi, Georgia, and the evolutionary biology of early Homo. Science. 2013;342(6156):326–31. doi: 10.1126/science.1238484 24136960

[8] Rightmire GP, Lordkipanidze D, Vekua A. Anatomical descriptions, comparative studies and evolutionary significance of the hominin skulls from Dmanisi, Republic of Georgia. J Hum Evol. 2006;50(2):115–41. doi: 10.1016/j.jhevol.2005.07.009 16271745

[9] Martinón-Torres M, Bermúdez de Castro JM, Gómez-Robles A, Margvelashvili A, Prado L, Lordkipanidze D, et al. Dental remains from Dmanisi (Republic of Georgia): morphological analysis and comparative study. J Hum Evol. 2008;55(2):249–73. doi: 10.1016/j.jhevol.2007.12.008 18486183

[10] Bermúdez de Castro JM, Martinón-Torres M, Sier MJ, Martín-Francés L. On the variability of the Dmanisi mandibles. PLoS One. 2014;9(2):e88212. doi: 10.1371/journal.pone.0088212 24586309 PMC3930530

[11] Alves Neves W, Vicensotto Bernardo D. The first hominin of Europe. Rev Arqueol. 2011;24(1):102–10. doi: 10.24885/sab.v24i1.317

[12] Lordkipanidze D, Vekua A, Ferring R, Rightmire GP, Agusti J, Kiladze G, et al. Anthropology: the earliest toothless hominin skull. Nature. 2005;434(7034):717–8. doi: 10.1038/434717b 15815618

[13] Lordkipanidze D, Vekua A, Ferring R, Rightmire GP, Zollikofer CPE, Ponce de León MS, et al. A fourth hominin skull from Dmanisi, Georgia. Anat Rec A Discov Mol Cell Evol Biol. 2006;288(11):1146–57. doi: 10.1002/ar.a.20379 17031841

[14] Skinner MM, Gordon AD, Collard NJ. Mandibular size and shape variation in the hominins at Dmanisi, Republic of Georgia. J Hum Evol. 2006;51(1):36–49. doi: 10.1016/j.jhevol.2006.01.006 16563468

[15] Scardia G, Neves WA, Tattersall I, Blumrich L. What kind of hominin first left Africa? Evol Anthropol. 2021;30(2):122–7. doi: 10.1002/evan.21863 32893976

[16] Neves W, Senger MH, Valota L, Hubbe M. Revisiting the cranial variability of the Dmanisi hominins. Anthropol Rev. 2024;87(2):113–25.

[17] Schwartz JH, Tattersall I, Chi Z. Comment on “A complete skull from Dmanisi, Georgia, and the evolutionary biology of early Homo”. Science. 2014;344(6182):360. doi: 10.1126/science.1250056 24763572

[18] Guimarães SWF, Merino CL. Dmanisi hominin fossils and the problem of the multiple species in the early Homo genus. Nexus Can Stud J Anthropol. 2015;23(2):1–21. doi: 10.15173/nexus.v23i2.894

[19] Argue D, Bermúdez deCastro JM, Lee MSY, Martinón-Torres M. Where do the Dmanisi hominins fit on the human evolutionary tree? bioRxiv. 2025.

[20] Smith TM. Teeth and human life-history evolution. Annu Rev Anthropol. 2013;42:191–208.

[21] Guatelli-Steinberg D. What teeth reveal about human evolution. Cambridge: Cambridge University Press; 2016.

[22] Suwa G, Wood BA, White TD. Further analysis of mandibular molar crown and cusp areas in Pliocene and early Pleistocene hominids. Am J Phys Anthropol. 1994;93(4):407–26. doi: 10.1002/ajpa.1330930402 8048464

[23] Wood BA, Abbott SA. Analysis of the dental morphology of Plio-pleistocene hominids. I. Mandibular molars: crown area measurements and morphological traits. J Anat. 1983;136(Pt 1):197–219. 6403498 PMC1171940

[24] Wood BA, Abbott SA, Graham SH. Analysis of the dental morphology of Plio-Pleistocene hominids. II. Mandibular molars—study of cusp areas, fissure pattern and cross sectional shape of the crown. J Anat. 1983;137(Pt 2):287–314. 6415025 PMC1171822

[25] Wood BA, Uytterschaut H. Analysis of the dental morphology of Plio-Pleistocene hominids. III. Mandibular premolar crowns. J Anat. 1987;154:121–56. 3128512 PMC1261842

[26] Wood BA, Abbott SA, Uytterschaut H. Analysis of the dental morphology of Plio-Pleistocene hominids. IV. Mandibular postcanine root morphology. J Anat. 1988;156:107–39. 3047096 PMC1261917

[27] Wood BA, Engleman CA. Analysis of the dental morphology of Plio-Pleistocene hominids. V. Maxillary postcanine tooth morphology. J Anat. 1988;161:1–35. 3254883 PMC1262088

[28] Irish JD. Biological affinities of late Pleistocene through modern African aboriginal populations: the dental evidence. Arizona State University; 1993.

[29] Irish JD, Guatelli-Steinberg D. Ancient teeth and modern human origins: an expanded comparison of African Plio-Pleistocene and recent world dental samples. J Hum Evol. 2003;45(2):113–44. doi: 10.1016/s0047-2484(03)00090-3 14529648

[30] Berg GE, Ta’ala SC, editors. Size matters: discrimination between American Blacks and Whites, males and females, using tooth crown dimensions. In: Biological affinity in forensic identification of human skeletal remains. Boca Raton (FL): CRC Press; 2015. p. 209–38.

[31] Pilloud MA, Hefner JT, Hanihara T, Hayashi A. The use of tooth crown measurements in the assessment of ancestry. J Forensic Sci. 2014;59(6):1493–501. doi: 10.1111/1556-4029.12540 25060236

[32] Czelusniak J, Goodman M. Hominoid phylogeny estimated by model selection using goodness of fit significance tests. Mol Phylogenet Evol. 1995;4(3):283–90. doi: 10.1006/mpev.1995.1025 8845964

[33] Schneider H, Sampaio I, Harada ML, Barroso CML, Schneider MPC, Czelusniak J, et al. Molecular phylogeny of the New World monkeys (Platyrrhini, primates) based on two unlinked nuclear genes: IRBP intron 1 and ε-globin sequences. Am J Phys Anthropol. 1996;100(2):153–79. doi: 10.1002/(sici)1096-8644(199606)100:2<153::aid-ajpa1>3.0.co;2-z 8771309

[34] Page SL, Chiu C, Goodman M. Molecular phylogeny of Old World monkeys (Cercopithecidae) as inferred from gamma-globin DNA sequences. Mol Phylogenet Evol. 1999;13(2):348–59. doi: 10.1006/mpev.1999.0653 10603263

[35] Page SL, Goodman M. Catarrhine phylogeny: noncoding DNA evidence for a diphyletic origin of the mangabeys and for a human-chimpanzee clade. Mol Phylogenet Evol. 2001;18(1):14–25. doi: 10.1006/mpev.2000.0895 11161738

[36] Irish JD, Grabowski M. Relative tooth size, Bayesian inference, and Homo naledi. Am J Phys Anthropol. 2021;176(2):262–82. doi: 10.1002/ajpa.24353 34190335

[37] Berger LR, de Ruiter DJ, Churchill SE, Schmid P, Carlson KJ, Dirks PHGM, et al. Australopithecus sediba: a new species of Homo-like australopith from South Africa. Science. 2010;328(5975):195–204. doi: 10.1126/science.1184944 20378811

[38] Strait DS, Grine FE. Inferring hominoid and early hominid phylogeny using craniodental characters: the role of fossil taxa. J Hum Evol. 2004;47(6):399–452. doi: 10.1016/j.jhevol.2004.08.008 15566946

[39] Scott JE, Lockwood CA. Patterns of tooth crown size and shape variation in great apes and humans and species recognition in the hominid fossil record. Am J Phys Anthropol. 2004;125(4):303–19. doi: 10.1002/ajpa.10406 15386248

[40] Neves W, Valota L. Evolução hominínia: o que revela o tamanho dos dentes posteriores? Rev Arqueol. 2024;37(3):3–25. doi: 10.24885/sab.v37i3.1191

[41] Pilloud MA, Adams DM, Hefner JT. Observer error and its impact on ancestry estimation using dental morphology. Int J Legal Med. 2019;133(3):949–62. doi: 10.1007/s00414-018-1985-3 30564914

[42] Hubbe M, Harvati K, Neves W. Paleoamerican morphology in the context of European and East Asian late Pleistocene variation: implications for human dispersion into the New World. Am J Phys Anthropol. 2011;144(3):442–53. doi: 10.1002/ajpa.21425 21302270

[43] R Core Team. R: a language and environment for statistical computing. Vienna, Austria: R Foundation for Statistical Computing; 2024.

[44] Venables WN, Ripley BD. Modern applied statistics with S. 4th ed. Springer; 2002.

[45] Wickham H. ggplot2: Elegant graphics for data analysis. Springer; 2016.

[46] Slowikowski K. ggrepel: Automatically position non-overlapping text labels with ‘ggplot2’. R package version 0.9.3; 2023.

[47] Zollikofer CPE, Beyrand V, Lordkipanidze D, Tafforeau P, Ponce de León MS. Dental evidence for extended growth in early Homo from Dmanisi. Nature. 2024;635(8040):906–11. doi: 10.1038/s41586-024-08205-2 39537931 PMC11602720

[48] Wood B. Did early Homo migrate “out of” or “in to” Africa? Proc Natl Acad Sci U S A. 2011;108(26):10375–6. doi: 10.1073/pnas.1107724108 21677194 PMC3127876

[49] Ferring R, Oms O, Agustí J, Berna F, Nioradze M, Shelia T. Earliest human occupations at Dmanisi (Georgian Caucasus) dated to 1.85–1.78 Ma. Proc Natl Acad Sci U S A. 2011;108(26):10432–6. 21646521 10.1073/pnas.1106638108 PMC3127884

[50] Carotenuto F, Tsikaridze N, Rook L, Lordkipanidze D, Longo L, Condemi S, et al. Venturing out safely: the biogeography of Homo erectus dispersal out of Africa. J Hum Evol. 2016;95:1–12. doi: 10.1016/j.jhevol.2016.02.005 27260171

[51] Agustí J, Lordkipanidze D. How “African” was the early human dispersal out of Africa? Quat Sci Rev. 2011;30(11–12):1338–42.

[52] Agustí J, Lordkipanidze D. An alternative scenario for the first human dispersal out of Africa. L’Anthropologie. 2019;123(4–5):682–7. doi: 10.1016/j.anthro.2019.102727

[53] Husson L, Salles T, Lebatard AE. Javanese Homo erectus on the move in SE Asia circa 1.8 Ma. Sci Rep. 2022;12:19012. 36347897 10.1038/s41598-022-23206-9 PMC9643487

[54] Luo L, Granger DE, Tu H, Lai Z, Shen G, Bae CJ, et al. The first radiometric age by isochron 26Al/10Be burial dating for the Early Pleistocene Yuanmou hominin site, southern China. Quat Geochronol. 2020;55:101022.

[55] Palmqvist P, Rodríguez-Gómez G, Figueirido B, García-Aguilar JM, Pérez-Claros JA. On the ecological scenario of the first hominin dispersal out of Africa. L’Anthropologie. 2022;126(1):102998. doi: 10.1016/j.anthro.2022.102998

[56] Parenti F, Varejão FG, Scardia G, Okumura M, Araujo A, Ferreira Guedes CC, et al. The Oldowan of Zarqa Valley, Northern Jordan. J Paleolit Archaeol. 2024;7(1):3.

[57] Curran SC, Drăgușin V, Pobiner B, Pante M, Hellstrom J, Woodhead J, et al. Hominin presence in Eurasia by at least 1.95 million years ago. Nat Commun. 2025;16(1):836. doi: 10.1038/s41467-025-56154-9 39833162 PMC11747263

